# Open-Source 3D Printable GPS Tracker to Characterize the Role of Human Population Movement on Malaria Epidemiology in River Networks: A Proof-of-Concept Study in the Peruvian Amazon

**DOI:** 10.3389/fpubh.2020.526468

**Published:** 2020-09-24

**Authors:** Gabriel Carrasco-Escobar, Kimberly Fornace, Daniel Wong, Pierre G. Padilla-Huamantinco, Jose A. Saldaña-Lopez, Ober E. Castillo-Meza, Armando E. Caballero-Andrade, Edgar Manrique, Jorge Ruiz-Cabrejos, Jose Luis Barboza, Hugo Rodriguez, German Henostroza, Dionicia Gamboa, Marcia C. Castro, Joseph M. Vinetz, Alejandro Llanos-Cuentas

**Affiliations:** ^1^Health Innovation Laboratory, Institute of Tropical Medicine “Alexander von Humboldt”, Universidad Peruana Cayetano Heredia, Lima, Peru; ^2^Division of Infectious Diseases, Department of Medicine, University of California, San Diego, La Jolla, CA, United States; ^3^Laboratorio ICEMR-Amazonia, Laboratorios de Investigación y Desarrollo, Facultad de Ciencias y Filosofía, Universidad Peruana Cayetano Heredia, Lima, Peru; ^4^Faculty of Infectious and Tropical Diseases, London School of Hygiene and Tropical Medicine, London, United Kingdom; ^5^Departamento de Ingenieria, Facultad de Ciencias y Filosofía, Universidad Peruana Cayetano Heredia, Lima, Peru; ^6^Dirección Regional de Salud Loreto, Loreto, Peru; ^7^Department of Medicine, University of Alabama at Birmingham, Birmingham, AL, United States; ^8^Departamento de Ciencias Celulares y Moleculares, Facultad de Ciencias y Filosofía, Universidad Peruana Cayetano Heredia, Lima, Peru; ^9^Instituto de Medicinal Tropical Alexander von Humboldt, Universidad Peruana Cayetano Heredia, Lima, Peru; ^10^Department of Global Health and Population, Harvard T. H. Chan School of Public Health, Boston, MA, United States; ^11^Section of Infectious Diseases, Yale School of Medicine, New Haven, CT, United States; ^12^Facultad de Salud Pública y Administración, Universidad Peruana Cayetano Heredia, Lima, Peru

**Keywords:** malaria, human movement, movement ecology, asymptomatic malaria, connectivity, networks, Amazon, open-source

## Abstract

Human movement affects malaria epidemiology at multiple geographical levels; however, few studies measure the role of human movement in the Amazon Region due to the challenging conditions and cost of movement tracking technologies. We developed an open-source low-cost 3D printable GPS-tracker and used this technology in a cohort study to characterize the role of human population movement in malaria epidemiology in a rural riverine village in the Peruvian Amazon. In this pilot study of 20 participants (mean age = 40 years old), 45,980 GPS coordinates were recorded over 1 month. Characteristic movement patterns were observed relative to the infection status and occupation of the participants. Applying two analytical animal movement ecology methods, utilization distributions (UDs) and integrated step selection functions (iSSF), we showed contrasting environmental selection and space use patterns according to infection status. These data suggested an important role of human movement in the epidemiology of malaria in the Peruvian Amazon due to high connectivity between villages of the same riverine network, suggesting limitations of current community-based control strategies. We additionally demonstrate the utility of this low-cost technology with movement ecology analysis to characterize human movement in resource-poor environments.

## Introduction

The Loreto Department of Peru, in the Amazon region, is the most important malaria-endemic area of the country where more than 95% of country-wide cases are transmitted. Most of these cases are due to *Plasmodium vivax* (80%), followed by *P. falciparum* (20%), and are mainly transmitted by *Nyssorhynchus* (*Anopheles) darlingi*. Intensified control efforts targeted to high incidence villages facilitated a reduction in malaria in the 2006–2010 period (1). From 2011 to 2017, however, a dramatic increase in the number of malaria cases has been observed in Loreto. Although the large reservoir of asymptomatic infections and decreasing political commitment toward malaria control likely contributed to this resurgence, the impact of human movement between areas of differing malaria transmission remains poorly understood.

In the Peruvian Amazon, malaria transmission is complex, occurring both in villages and along river networks with dense forest coverage where occupation-related mobility brings infected people into proximity with vector mosquitoes (1–3). In these settings, highly heterogeneous patterns have been observed in malaria infections and vector indexes (3–7). Although an intense flow of parasite populations in these areas support the hypothesis of high connectivity between villages at micro-geographic scales (8–10), the magnitude and impact of human movement in malaria epidemiology is poorly understood.

Human movement has been shown to affect malaria epidemiology at multiple geographical levels (11–13), and recent technological advances using mobile phones and GPS tracking have led to new insights into fine-scale behaviors and movement processes (14–18). However, environmental conditions and lack of service networks (telephone, internet, etc.) prevents the use of mobile cell phone technologies in rural Amazonia. Alternative approaches to geo-reference self-reported trajectories seem to be promising to overcome these limitations (19, 20). For example, self-reported movement patterns showed high connectivity among villages in a single watershed in Peru (20), consistent with previous studies based on travel questionnaires (3), and showed that travel outside the community was a risk factor for malaria (3, 4, 20, 21). Portable Global Positioning System (GPS) tracking devices have been utilized to collect detailed movement data in peri-urban and rural settings (22, 23). By applying movement ecology approaches, these data can be combined with spatial and environmental data to relate the probability of an individual using a particular space with characteristics of that location (24); these approaches have been applied to examine fine-scale movement into malaria vector habitats (25). However, they have not been applied within a riverine setting, and the costs of utilizing those devices at a population level (~100 USD per GPS tracker) remain prohibitively expensive for public health programs in this region.

These previous findings indicate potential importance of human mobility in the maintaining and spreading malaria transmission in river networks in the Peruvian Amazon and highlight the need for new tools to measure and characterize these movement patterns. As a consequence of this knowledge gap, the MoH remains focused on control activities in high-risk communities as single entities, instead of encompassing highly connected landscape units (i.e., communities within watersheds). This study addresses this gap and aimed to develop a new device to monitor and describe human movement patterns, as a step to provide evidence of the role of human population movement on malaria epidemiology in rural villages in the Peruvian Amazon river networks. An open-source low-cost 3D printable GPS tracker was developed and manufactured, tailored for conditions in the watersheds, to collect fine-scale human movement. Additional information on basic demographics and malaria infection status was also collected. To identify potential risk factors for infection associated with mobility, novel movement ecology analytical approaches were applied to describe heterogeneities in movement patterns by socio-demographic characteristics and infection status of villagers.

## Methods

### Ethics

This study was approved by the Ethics Review Board of the Regional Health Directorate of Loreto and Universidad Peruana Cayetano Heredia in Lima. IRB approval number #100469. Participants were enrolled upon signed an informed consent. All the methods were carried out in accordance with the approved guidelines.

### Study Design

We conducted a proof-of-concept study to quantify human population movement in a riverine community, and its relative contribution to malaria epidemiology in complex river networks, the most common setting in the Peruvian Amazon. An open-source 3D-printable GPS tracker was developed for this context (no mobile or internet network, dense cloud coverage, movements through water environments) and a weekly cohort was carried out on June 04-27, 2018.

### Study Site and Population

**Figure 1:**
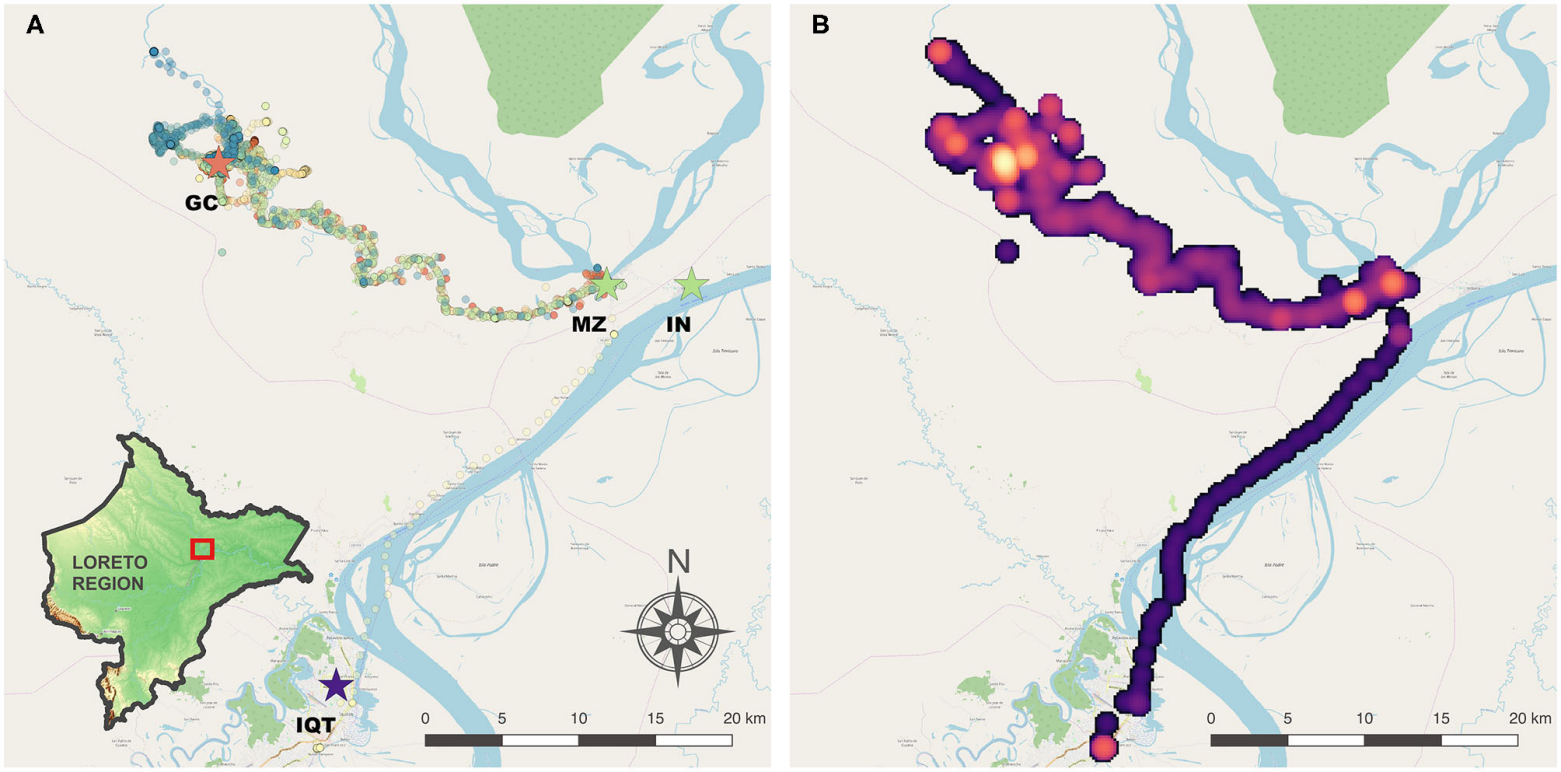
Study area in Mazan district, Loreto Region, Peruvian Amazon. **(A)** GPS tracks collected and location of Gamitanacocha (GC), main ports: Mazan (MZ) and Indiana (IN), and Iquitos Capital City (IQT). Each color represents a participant. **(B)** Heatmap of transit based on GPS tracks. Maps were produced using QGIS 2.16 (QGIS Development Team, 2018. QGIS Geographic Information System. Open Source Geospatial Foundation Project. https://www.qgis.org/) based on public geographic data extracted from © OpenStreetMap contributors (www.openstreetmap.org) under Open Data Commons Open Database License (ODbL) 1.0 (http://openstreetmap.org/copyright).

This study was carried out in Gamitanacocha (3.426°S, 73.318°W), district of Mazan, province of Maynas in the Loreto Region (Figure 1). This village is located in the Mazan River (north of Iquitos city, capital of Loreto) and is only reachable by boat (~ 6 h from Iquitos City). The landscape is composed by dense primary and secondary tropical forest, with a rainy season between November and May, and a dry season between June and October. This ecological setting is highly suitable for *Nyssorhynchus* (*Anopheles*) *darling* breeding, the primary vector of malaria in this area (7, 26).

Gamitanacocha is a community classified as one of the villages with the highest risk of malaria transmission along the Mazan river with a population of 92 inhabitants (3). Previous studies have described complex malaria dynamics in this area associated with occupational-related mobility (3, 4). Twenty GPS devices were manufactured for this proof-of-concept study. Descriptive comparison between participants with different infection status was aimed, although no statistical tests were intended due to small sample size. A purposive sampling of 20 participants (40% of inhabitants aged 18 years old or above in the community) was carried out proportional on whether the participant self-reported a travel in the previous month at the baseline (Supplementary Table 1).

### Device Development

For this study, the GPS-tracker was developed and manufactured tailored for conditions in the Amazon rainforest. Main characteristics includes: (1) Long-life battery (to collect data of long-duration trajectories); (2) Capacity to store the GPS coordinates and timestamp, as well as other key characteristics such as the number of satellites, battery life status, quality of the GPS signal, and assigned participant ID; (3) Set a community boundary (i.e., study area) and report whether the participant moved outside that boundary, herein referred to as location status; (4) Block configuration commands, so that only the research team can configure the settings of the device; (5) Easy set-up in the field using a Laptop or Tablet. Configurable features include participant ID label, community boundary (i.e., centroid and radius), time interval for GPS collection and time duration for active and sleep modes, tolerance of GPS error (based on a predefined boundary area or, otherwise, on a distance from previous correct GPS coordinate).

### Hardware Architecture and Code Description

Full description of development and manufacturing are presented in Supplementary Methods and printable files are available at https://github.com/healthinnovation/gorgas_tracker.

**Figure 2:**
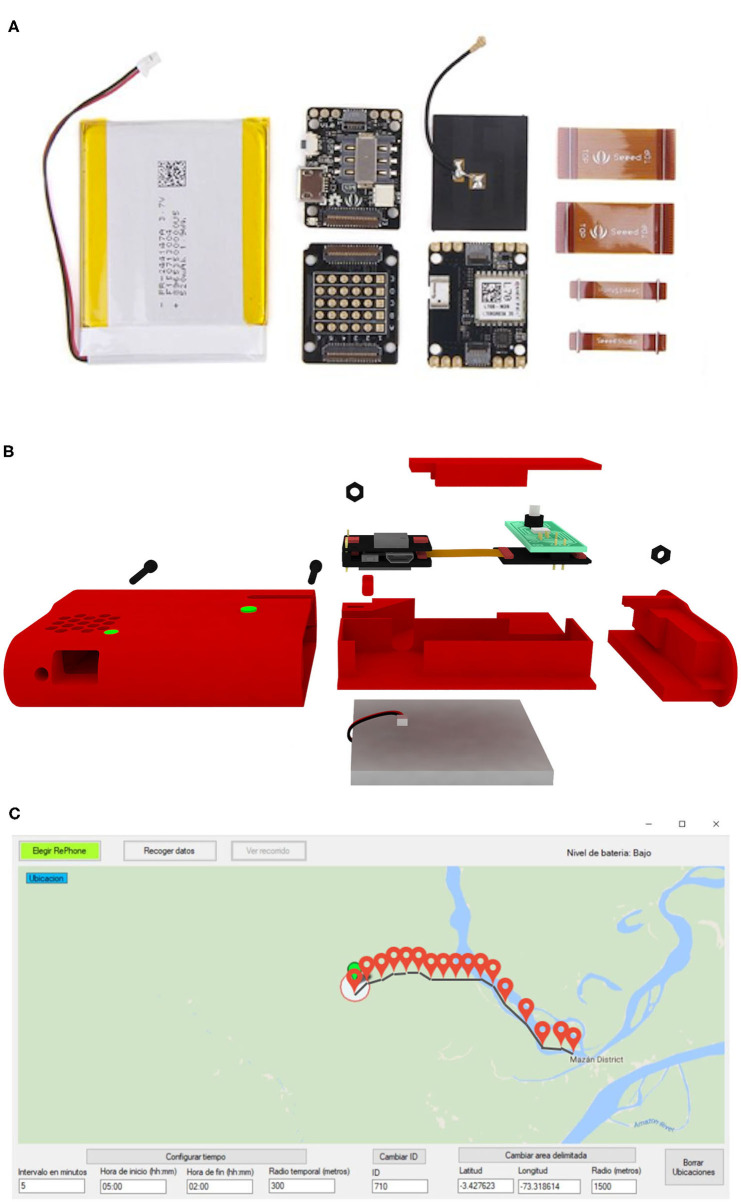
Open-source 3D printable GPS-tracker design and development. **(A)** SeeedStudio-RePhone modules used for the construction of the device. **(B)** CAD 3D model of the GPS-tracker device. **(C)** Preview of the configuration tool. Map shown in the configuration tool was produced using QGIS 2.16 (QGIS Development Team, 2018. QGIS Geographic Information System. Open Source Geospatial Foundation Project. https://www.qgis.org/) based on public geographic data extracted from © OpenStreetMap contributors (www.openstreetmap.org) under Open Data Commons under Open Data Commons Open Database License (ODbL) 1.0 (http://openstreetmap.org/copyright).

Briefly, the GPS-tracker device was based on an open hardware platform called RePhone, designed by SeeedStudio as a new form of phone customization and a wearable/IoT development board (27). RePhone Geo Kit was selected due to the size of the modules and key features (e.g., real-time geographic position, traveling speed and time information) including its capacity to track 22 satellites in 66 channels (Figure 2A).

An iterative case (container of the modules) design was conducted. Computer-aided design (CAD) models of 6 case prototypes are presented in Supplementary Figure 1. Each iteration was made in order to fulfill the requirements of user interaction, portability, and efficient tracking. The final model included a bigger battery space and efficient printing of spare parts (Figure 2B). An efficient power management algorithm was developed to achieve more than 6 days of battery life (Supplementary Methods 1.1).

In order to inspect the report of whether the participant had moved outside the boundaries of the community, a new module with RGB light-emitting diodes (LED) and two buttons, and an algorithm were designed. The color code was red when the participant moved outside the boundaries, green when they did not register movement outside the boundaries, and blue when incorrect GPS data was recorded at initialization. Importantly, LED lights can only be activated by the research team to avoid modifications in the participant's movement.

The GPS-tracker algorithm workflow is described in Supplementary Methods 1.2. A user interface was developed for the sole use of the research team in a Laptop or Tablet via a USB connection. A graphic interface was developed on Visual Studio to visualize these datasets in a comprehensive way (Figure 2C). The GPS-tracker baseline characteristics and configuration were described in Supplementary Methods 1.3.

### Epidemiologic and Socio-Demographic Data

At the baseline, all villagers > 18 y (*n* = 50) self-reported whether they traveled in the previous month (sample stratification criteria), sex, age, migrant status (whether they were born in a different village), and occupation. All occupation activities were classified into two major groups according to where the activities were carried out (inside or outside the community). Loggers, fishermen, and traders were classified as out-community occupation and all the others as in-community occupations. Previous studies (3) in this area showed that ~85% of infections are submicroscopic (undetectable by microscopy but detectable by molecular inspection), thus, to account for the overall burden of malaria infections, the infection status was assigned according to molecular (PCR) diagnostic at *Plasmodium* spp. genus level. Participants were classified as infected if they had a positive diagnostic at least at once during the study period, or non-infected otherwise.

### Human Movement Data

Upon enrollment, a unique code was assigned to each participant and paired to the GPS-tracker device. A description of the functions and use of the GPS-tracker devices were provided to the participants by the fieldwork team. Also, a water-proof case was provided with a buckle so they can carry it in the arm, belt or pocket. During each weekly visit, the battery of the GPS-tracker was replaced to maximize the activity period of the devices and participants were screened and incentivized to ensure compliance for device use. In addition, a standardized travel questionnaire was conducted and compared with the GPS-tracker device.

### Blood Sample Collection

Blood samples on filter paper for PCR diagnostic were collected by finger-prick if: (1) the participant self-reported travel outside the village; (2) the GPS-tracker recorded travel outside the village; (3) the participant presented clinical symptoms compatible with malaria. A second blood sample was collected after 4 days to avoid misclassification of infection status due to disease progression (undetectable parasitemias at the beginning of the infection). *Plasmodium* species-specific identification was conducted using a modified Mangold et al. (28) protocol. Further details in Supplementary Methods 1.4.

### Data Processing and Analyses

Fisher's exact test for categorical variables and *t*-test for continuous variables were used for significance testing between villagers in Gamitanacocha that were included (*n* = 20) and not included (*n* = 30) in this study.

A trajectory was defined as a single person-day follow-up. Geo-processing and trajectory statistics were described in Supplementary Methods 1.5. To characterize the total amount of space used by participants in addition to the distance traveled, we calculated the utilization distribution (UD) for each individual, the probability of an individual being in a specific location during the sampled time. Within movement ecology, UDs are used to estimate the size of a home range and characterize the frequency different areas are used; these approaches have also been applied to understand space use of hunter gather populations (24). UDs were estimated for each individual using biased random bridges (BRBs) (29). In contrast to kernel smoothing methods, BRBs estimate UDs using a time-ordered series of points, allowing interpolation of missing values and adjustment for spatial error and irregular sampling times. To fit BRBs, we estimated the maximum time at which movements were uncorrelated as 3 h, the minimum distance below which an individual is considered stationary as 10 m and the minimum standard deviation in relocation uncertainty as 30 m. These parameters were chosen to account for GPS recording error as well as typical movement patterns within this region. Estimates of home range use were based on the 95th percentile, representing the area with a 95% cumulative probability distribution of use by the individual during the sampling period. We additionally evaluated the home range core area, the area most commonly used by the individual, based on the 50th percentile. We analyzed data separately for each individual for all recorded movements and for only movements occurring during peak mosquito biting periods (6 P.M.−6 A.M).

Travel categories (movement profiles) were derived from travel distance and duration, and the infection status associated with each timestamp. Each travel person-day were then grouped and compared according to occupation categories. To understand the environmental selection process by villagers moving through the landscape, an integrated step-selection function (iSSF) analysis was conducted with the “amt” package in R (30). iSSF are commonly used to link environmental covariates to animal tracks, however in this study was used to make inference regarding environmental selection and movement processes along a Euclidean gradient from their home village comparing between infection status. Briefly, iSSF are estimated by comparing observed steps connecting successive locations to random steps, using a likelihood equivalent of a Cox proportional hazards model (30, 31). The underlying surface was constructed based on a raster with the Euclidean distance from Gamitanacocha and categorized in 10 equal-length intervals. A separate iSSF analysis was conducted for each infection status. We demonstrate how this data can be visualized to explore associations of risk between either individual characteristics or specific movements or trips.

Maps were generated with QGIS 2.18 (QGIS Geographic Information System, Open Source Geospatial Foundation Project: http://www.qgis.org) and all analyses and visualizations were produced using R v.3.6.0 (R Development Core Team, R Foundation for Statistical Computing, Australia).

## Results

### Baseline Characteristics

This study enrolled 20 participants between 18 and 77 (mean = 40) years old. Most (80%) self-reported travel within the previous year, following the same proportion of travel-reporting at the total population in the riverine community of Gamitanacocha (sample-stratification criteria). The most common occupations (logging, farming). take place outside the community (70%); and 40% of participants were migrants (born in a different village). A higher proportion of males (75%) was observed in the sample in comparison to the general population (56%). Gender and study level were the only variables with statistically significant differences between participants included and non-included in the study. Malaria infection status among the enrolled villager was comparable to those not enrolled. Most (60%) were infected at least once during the 1-month follow-up period (Supplementary Table 1).

### Human Movement Records

After initial cleaning, 45,980 GPS coordinates were processed (Figure 1A). Movement tracking of 256 person-days were collected (56% of total follow-up). The boundary box of the human movements encompassed ~2,150.900 km^2^. As expected, most of the movements were recorded around Gamitanacocha village boundaries, however important areas were detected in neighboring villages, and the Mazan city, capital of the Mazan district and main port toward the Amazon River and Iquitos City (Figure 1B). As expected, the number of coordinates, step length, total length, and duration of the follow-up were comparable between infected and non-infected participants. Important differences were observed in the expected square displacement, the duration of travels, and distance of travels according to infection status with greater values observed in infected compared with non-infected participants. The variability was also greater in infected in comparison with non-infected participants (Table 1).

**Table 1:** Human population movement descriptive statistics.

**Status**	**Person-day follow-up**	**Number of coordinates**		**Step length (Km)**		**Total length (Km)**		**Duration follow-up (h)**		**Expected square displacement (Km)**		**Duration travel (h)**		**Distance travel (Km)**	
		**Mean**	**(sd)**	**Mean**	**(sd)**	**Mean**	**(sd)**	**Mean**	**(sd)**	**Mean**	**(sd)**	**Mean**	**(sd)**	**Mean**	**(sd)**
Non-infected	101	178.55	50.53	0.035	0.054	6.456	10.84	20.31	3.953	5.1662	16.427	8.207	5.804	1.9937	5.3064
Infected	155	180.30	52.28	0.042	0.069	7.642	13.03	20.25	4.19	9.7455	28.824	9.122	6.549	2.8332	7.4349

### Infection Status

Of the 20 participants followed, 11 (55%) recorded long-distance movements (>5 km), 7 (35%) recorded maximum movements between neighboring villages, and only 2 (10%) stayed in the village during all the study period. In addition, single infection 7-day windows aligned with participant's movements are shown in Supplementary Figure 2. Out of the 12 infected participants, 4 (33%) carried out a long-distance movement during the infection period (infection carriers), 3 (25%) carried out movements to neighboring villages, and 5 (42%) remained in the community or farms during the infection period. From the 11 participants recording long-distance movements along the study period, the infection period encompasses only movements within Gamitanacocha in 3 of them, movements between neighboring villages in one of them, long-distance movements in 4 of them, and 3 were not infected during the movements along the study period.

### Movement Profiles

**Figure 3:**
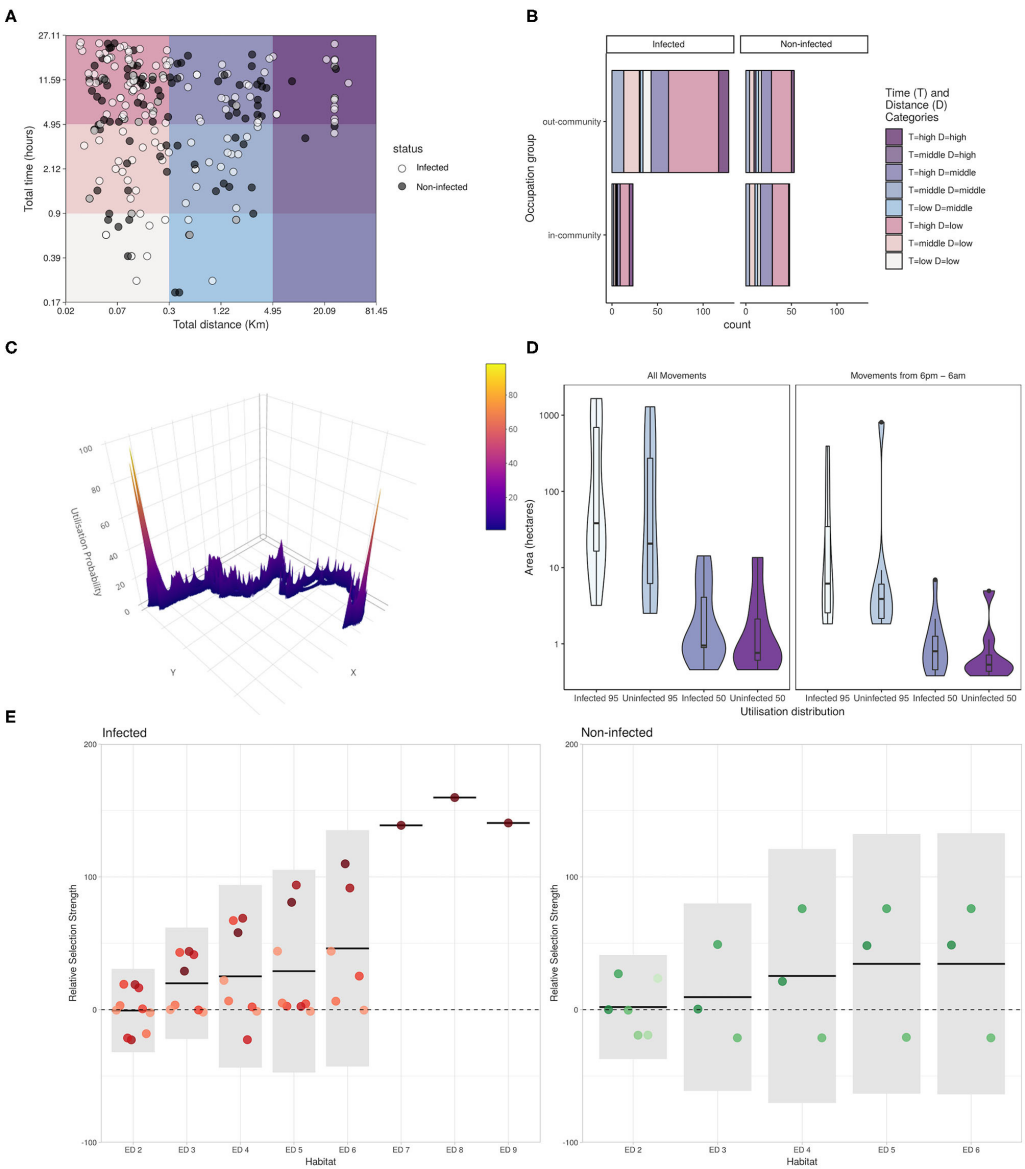
Mobility patterns, Utilization Distributions (UDs) and Integrated Step-Selection Functions (iSSF) of Euclidean distance (ED) categories relative to infection status of inhabitants of Gamitanacocha. **(A)** Distribution of profiles among categories of travel distance and time (X- and Y- axes in logarithmic scale). Each point represents a trajectory (person-day travel) and colors represent infection status (dark = non-infected, white = Infected). **(B)** Travel patterns relative to infection status and occupation activities. UDs estimates based on the cumulative probability of a Kernel Distribution (KD) at different percentiles. **(C)** Individual utilization distribution calculated from GPS tracks. **(D)** Sample distribution of core (50) and home (95) range relative to infection status and time of movement. **(E)** iSSF, each color represents a participant. Solid horizontal lines represent the population-level estimates and 95% confidence intervals are given by the light gray boxes. The dashed horizontal line indicates no preference relative to Euclidian Distance (ED) category 1 [i.e., the community boundaries (the reference category)].

Marked movement patterns were observed in this study (Figure 3A). The most common pattern was short-distance movements (<300 m from Gamitanacocha) in long time periods (> 5 h). The infection status was scattered distributed across movement profiles, expected due to the 7-day window. Importantly, a high proportion of long-distance travel (> 5 km from Gamitanacocha) were carried out by infected participants, in comparison to short-distance movements where not a clear pattern was observed (Figure 3A). Interestingly, the greatest distances and periods were related to out-community occupational-related activities (Figure 3B). Infected villagers recorded more movements than non-infected villagers, and in addition, the out-community movements were remarkably higher in infected (by molecular test) than non-infected villagers (Figure 3B).

### iSSF and UD

This study used two analytical methods derived from the animal movement ecology literature: utilization distributions (UDs) and integrated step selection functions (iSSF). UDs describe the probability of an individual being in a particular location during the sampling period, based on the frequency of recorded visits to these locations and the mean residence time per visit (29). Home range estimates, areas with a cumulative probability of 95% based on the UD, varied substantially by individual, from 2.51 to 1,652.18 hectares (ha) (mean: 349.63 ha) for all movements and 1.83 to 807.62 ha (mean: 77.47 ha) for movements only between 6 P.M. and 6 A.M. Similarly, core home range estimates varied from 0.48 to 14.24 ha (mean: 3.13 ha) for all movements and 0.38 to 6.93 ha (mean: 1.29 ha) for night-time movements. The size of the area used was mostly higher for infected participants compared to uninfected participants (Figures 3C,D). While men tended to have larger home range estimates both during all movements and night-time movements, statistical comparisons could not be conducted due to the small sample size.

Using integrated step selection functions (iSSF) to examine relationships between spatial distance from Gamitanacocha and movement, Figure 3E shows the contrasting iSSF patterns between infected and non-infected participants. A marked increasing pattern was observed in infected participants along Euclidian distance categories from Gamitanacocha, however, due to the small sample size, confidence intervals included the null hypothesis. Conversely, non-infected participants showed a pattern most compatible with no environmental selection across distance categories.

## Discussion

This study provides a field-deployable approach to obtaining objective GPS data to characterize the role of human movement in the epidemiology of malaria in river networks in the Peruvian Amazon. Although human movement has previously been indirectly pointed as important for malaria risk and exposure based on epidemiologic, molecular and vector biology studies (3, 4, 8, 9), and also portrayed using new geo-referencing approached tailored to the lack of network accessibility in these settings (19, 20), this study provides the basis to obtain fine-scale resolution to obtain evidence regarding the influence of human movement in malaria transmission. This study additionally demonstrates the utility of a low-cost 3D printable GPS technology combined with movement ecology analytical approaches to collect and characterize human movement patterns for disease studies.

The preliminary findings shown here suggest a higher movement to other villages in the same watershed than previously proposed. This movement inevitably increases the *importation risk* (previously known as *vulnerability*) in settings with heterogeneous malaria transmissions (32), this in turn is a component of the *malariogenic potential*, that is defined as the “likelihood of local transmission that is the product of receptivity, risk of importation of malaria parasites and infectivity of imported parasites” (32). Thus, as the landscape of most malaria high-risk areas in Loreto are comparable to the river network analyzed in this study, the high connectivity between risk-heterogeneous units likely contributed to the rapid rebound of malaria transmission observed after the interruption of intervention coverage after 2010. However, as we only sampled participants from one village, further studies are needed to fully evaluate the connectivity between villages within this watershed, including movements of other populations into Gamitanacocha. Although this analysis clearly demonstrates the movement of pathogens and people to surrounding areas, further population-based longitudinal studies are needed to characterize the roles of these movements in malaria transmission.

These findings are consistent with previous studies in the Amazon region that used self-reported travel questionnaires (3) and participatory mapping (20). In comparison with the aforementioned approaches, custom GPS monitors reported in this study, were able to describe fine-scale resolution and a larger variety of movement patterns, and this approach has the advantage of avoiding recall bias. Trajectories confirmed that villagers with occupational activities outside the community were the most active movers, who were also those who presented more malaria infections. These preliminary results support the hypothesis of high importation risk due to occupational activities. A higher sample size along with genetic epidemiological data will be required to more confidently delineate travel patterns that might predict local and imported cases.

The movement of asymptomatic *Plasmodium* spp. carriers, regardless of the location of infection acquisition, represent an important barrier to current malaria control strategies in the Peruvian Amazon. These findings are consistent with previous molecular studies where high parasite population flow was observed at micro-geographical scales and was hypothesized to be due to high human movement (8–10). The concurrent flow of human and parasite populations potentially increases the diversity of strains with different drug-resistant profiles (33), genome deletion that prevents the diagnostic capacity (i.e., HRP-2 deletion) (34), and infectivity efficiency, defined as the “ability of a given Plasmodium strain to establish an infection in an *Anopheles* mosquito species and undergo development until the mosquito has sporozoites in its salivary glands”(32).

Movement ecology approaches shed light to the movement processes. Analysis of space use (as measured by UD) revealed substantial heterogeneity in individual movements, with infected individuals primarily using larger geographic areas. Importantly, similar trends were observed during peak mosquito biting times at night, suggesting interventions targeted only at households may not prevent malaria in these individuals. Results from these analyses demonstrate how low-cost GPS technology can be combined with movement ecology approaches to quantitatively estimate human space use and environmental selection for epidemiological studies. A substantial variation in environmental selection between individuals with different infection statuses was observed, although no significant differences in environmental selection between infected and non-infected individuals were observed as consequence of the small sample size and wide range. Of movement patterns for the same participant; however marked patterns were observed in infected participants than those non-infected during the study period.

Taken together, these findings suggest that malaria control efforts in Peruvian rural Amazon might prioritize not only high-risk units (villages or districts), but also include their highly connected units to address malaria importation or exportation during transit or return to those units. In this sense and taking into account the topography of the Peruvian Amazon, river basins arise as promising surveillance units. Further studies are suggested to assess the effectiveness of such surveillance units as new approach.

Despite fine spatio-temporal coverage of human movements in the study, some limitations were recognized in this study. First, it is recognized that human movement can vary seasonally (13), especially in the Peruvian Amazon where particular occupations, such as logging and fishing, depend on seasonal environmental conditions (3, 4). It is expected that villagers change their movement profiles to seek better conditions to carry out their activities. This study only recorded the human movement in 1-month surveillance and studies with longer surveillance periods could capture the changes in the movement profile relative to the changes in the malaria transmission. Secondly, despite field-workers replacing battery in the weekly visits, gaps in movement tracking were observed. While analytic approaches developed to address these sampling inaccuracies were applied (i.e., biased random bridges - BRBs), periods without data could potentially bias this study. Finally, the size and weight of the GPS tracker device are key characteristics for a routine use of these devices in the Loreto population (23). The device developed in this study has a larger size and weight than previously reported as ideal for these types of studies (23). Despite the fact that field-workers encouraged participants for the continuous use of the GPS tracking to minimize non-informative GPS tracks, few (~12.3 person-days of follow-up – 5% total follow-up period) non-moving periods were detected and cleaned during the geo-processing. Future work aimed to reduce the size and weight of the device but maintaining the battery life and characteristics described.

To conclude, this study developed an open-source low-cost 3D printable GPS tracker for epidemiological studies under challenging environmental conditions such as the Amazon jungle and provided evidence that suggest an important role of human movement in the epidemiology of malaria in river networks in the Peruvian Amazon. The collected fine-scale movement patterns were observed relative to participant's infection status and occupation activities. This evidence suggests that the commuting patterns between villages of the same river network potentially jeopardizes the current control strategies in these areas. Further studies are suggested to evaluate a more comprehensive watershed-based approach to improve the malaria control in the Peruvian Amazon river networks.

## Data Availability Statement

The datasets generated for this study are available on request to the corresponding author.

## Ethics Statement

The studies involving human participants were reviewed and approved by Ethics Review Board of the Regional Health Directorate of Loreto and Universidad Peruana Cayetano Heredia in Lima. IRB approval number #100469. The patients/participants provided their written informed consent to participate in this study.

## Author Contributions

GC-E, GH, HR, and AL-C conceived and designed the study. PP-H, JS-L, OC-M, and AC-A device development. EM, JR-C, DW, and JB supervised fieldwork. DG supervised the laboratory assays. GC-E and KF analyzed the data. GC-E, DG, JV, and AL-C funds acquisition. GC-E, KF, PP-H, MC, JV, and AL-C wrote the manuscript. All authors reviewed and approved the final manuscript.

## Conflict of Interest

The authors declare that the research was conducted in the absence of any commercial or financial relationships that could be construed as a potential conflict of interest.

## Supplementary Material

Analysis code is available on https://github.com/healthinnovation/gorgas_tracker.
