# Supplementary material for: Open-Source 3D Printable GPS Tracker to Characterize the Role of Human Population Movement on Malaria Epidemiology in River Networks: A Proof-of-Concept Study in the Peruvian Amazon
Source: Front Public Health. 2020 Sep 24;8:526468. doi: 10.3389/fpubh.2020.526468 (PMC7542225; doi:10.3389/fpubh.2020.526468)

Open-source 3D printable GPS tracker to characterize the role of human population movement on malaria epidemiology in river networks: A proof-of-concept study in the Peruvian Amazon.

Gabriel Carrasco-Escobar^1,2,3,*^, Kimberly Fornace^4^, Daniel Wong^3^, Pierre G. Padilla-Huamantinco^1,5^, Jose A. Saldaña-Lopez^5^, Ober E. Castillo-Meza^5^, Armando E. Caballero-Andrade^5^, Edgar Manrique^1,3^, Jorge Ruiz-Cabrejos^1,3^, Jose Luis Barboza^3^, Hugo Rodriguez^6^, German Henostroza^7^, Dionicia Gamboa^3,8,9^, Marcia C. Castro^10^, Joseph M. Vinetz^9,11^, Alejandro Llanos-Cuentas^9,12^

^1^Health Innovation Lab, Institute of Tropical Medicine “Alexander von Humboldt”, Universidad Peruana Cayetano Heredia, Lima, Peru

^2^Division of Infectious Diseases, Department of Medicine, University of California San Diego, La Jolla, CA, USA

^3^Laboratorio ICEMR-Amazonia, Laboratorios de Investigación y Desarrollo, Facultad de Ciencias y Filosofía, Universidad Peruana Cayetano Heredia, Lima, Peru.

^4^ Faculty of Infectious and Tropical Diseases, London School of Hygiene and Tropical Medicine, London, UK

^5^Departamento de Ingenieria, Facultad de Ciencias y Filosofía, Universidad Peruana Cayetano Heredia, Lima, Peru.

^6^Dirección Regional de Salud Loreto, Loreto, Peru.

^7^University of Alabama at Birmingham, Birmingham, United States of America.

^8^Departamento de Ciencias Celulares y Moleculares, Facultad de Ciencias y Filosofía, Universidad Peruana Cayetano Heredia, Lima, Peru

^9^Instituto de Medicinal Tropical Alexander von Humboldt, Universidad Peruana Cayetano Heredia, Lima, Peru

^10^Department of Global Health and Population, Harvard T.H. Chan School of Public Health, Boston, MA, USA

^11^Section of Infectious Diseases, Yale School of Medicine, New Haven, CT, United States of America

^12^Facultad de Salud Pública y Administración, Universidad Peruana Cayetano Heredia, Lima, Peru

*** Correspondence:**Gabriel Carrasco Escobar, MSc: [gabriel.carrasco@upch.pe](mailto:gabriel.carrasco@upch.pe)

**SUPPLEMENTARY INFORMATION**

1. **Supplementary Methods:**
   1. **Hardware architecture**

The proposed GPS-tracker device hardware consists of three small modules: control module, GPS module, and extension module (Fig. 6A). The control module has a MT2502 chip as the main component. This microcontroller unit is an ultra-small chip with integrated memory (4MB) and contains dual-mode Bluetooth and integrated 2G modem (1). MT2502 is Arduino IDE compatible (easing the coding) and has a power management unit. The GPS module is based on a GPS L70 component, which fully meets industrial standards, and combines an advanced assisted GPS called EASY™ (Embedded Assist System) with AlwaysLocate™ technology (2). This means that L70 technology can achieve fast positioning (even indoors) with low power consumption. This module can track up to 22 satellites on 66 channels with a high-sensibility receiver (-163dB tracking), and user accuracy within a 10 meters radius under the open sky. The extension module is a 30 breakout pads board that allows developers to extend RePhone functionalities as new sensors or interfaces. These spaced holes are connected directly to control module pins through a flexible flat (FPC) cable.

The power management feature from MT2502 was modified to work on two modes: active and sleep. Active mode allowed devices to collect data points on a specific time interval, while sleep mode turned off all functionalities to minimize battery use between the previously configured time. As a result, more than 6 days of battery life were achieved with a power source of 2500mAh.

The reporting module was coupled to the extension module allowing interaction with the control module in order to report the location status of the participant. The button interaction distribution was one tap to verify whether the participant moved outside the boundary (red or green), and two taps to verify the correct initialization (blue).

- 1. **Code description**

The GPS-tracker algorithm workflow has three stages: 1) GPS files reading; 2) Power management; and 3) GPS-tracking logs. The GPS files contain the data and metadata divided in four plain text files (.txt) files: “locations.txt” (containing the GPS coordinates, timestamp, number of satellites, battery life status, and quality of the GPS signal), “id.txt” (participant ID and location status), “perimeter.txt” (centroid and radius of the community), and “time.txt” (time interval and time for active and sleep modes). The GPS files reading stage starts with algorithm reading the id file to switch the led (red or green) on as an initial state. Then, the time file is read to set up times for power management and the time interval for tracking. Power management stage configures internal time and date variables from GPS data received. The code is always comparing the internal clock and date with the time and date collected from satellites system. If variables are correct, power management allows the control module to collect location and store data on the locations file. Power mode (time file) is read by the power management to switch data logger off or restore its operations. Finally, GPS-tracking logs collects participants location during the follow-up and constantly verify if a participant is outside the village boundary. As result update the location status in the id file.

- 1. **GPS baseline characteristics and configuration**

The dimensions of the final version of the device were 65.73 x 22.75 x 86.10mm and a weight of 104.7 g (Fig. 6B). The initial parameters of the GPS device were configured as follows: centroid of community boundary, radius: 2,000 meters, start of sleep mode 2:00 am, end of sleep mode 5:00 am, time interval of GPS collection: 6 minutes, distance from previous correct GPS coordinate (if validation is required): 1,500 meters. The final price to construct this device was $35 per unit.

- 1. **Laboratory procedures**

Genomic DNA was extracted from dry blood spots of ~6 mm2 sections using E.Z.N.A.® Blood DNA Kit (Omega Bio-tek®, USA), according to the manufacturer guidelines with slight modifications – addition of TEN (20 mM Tris-HCl, pH 8.0; 2 mM EDTA, pH 8.0; 0.2 M NaCl) buffer, supplemented with SDS 10% w/v. Subsequent amplification was done by a real-time quantitative PCR (qPCR) method targeting the 18SSU rRNA gene region of the *Plasmodium* species-specific region. Oligonucleotides 5-TAACGAACGA- GATCTTAA-3 and 5-GTTCCTCTAAGAAGCTTT-3 were used as primers as reported by Mangold et al. (3) and a modified protocol was used including PerfeCta SYBR Green Fast Mix (Quanta Biosciences, MD, USA). Ambiguous diagnostic results were confirmed by using a nested ssPCR method described elsewhere (4).

- 1. **Geo-processing and Trajectory statistics**

Geo-processing was conducted in R and all out-of-range coordinates (boundary box of the study area in Fig. 1A) were cleaned and then transformed to tracking data format with EPSG:32718, WGS 84/UTM zone 18S projection. Movement patterns were initially explored using a heatmap with a radius of 1000m was constructed using the GPS point layers. The infection status was aligned to a 7-days window in the timestamp of the GPS tracks to describe the movement of *Plasmodium spp.* carriers during the study period. This time window was selected to reflect the exoerythrocytic infectiousness period of the host, the interval of the incubation period where the participant could potentially transmit gametocytes to *Anophelines* before getting detected by the PCR diagnostic. This was also the time difference between visits in the study.

Trajectory statistics were computed with the ‘trajr’ package in R (5) where each trajectory correspond to a single person-day follow-up. Briefly, a trajectory was defined as a series of steps, each comprised of a length (Li), a turning angle (Δi), and a time, which together represents a simplification of a real path travelled by a participant. The expected square displacement was computed using the formula in Kareiva & Shigesada, 1983 (6). Total length and the duration of the trajectories were computed for each person-day, which represents the total path of the participants in a single day regardless of their movements. This means that the same total length and duration could be reached by participants that stay in the community or those that traveled outside the community. To account for this difference, a trip was defined as the transit to and return from the most distant point reached from Gamitanacocha. A raster with the Euclidean distance from Gamitanacocha was constructed to compute the furthest point in each trip. The difference in distance and timestamp from the origin to the most distant point were defined as the travel distance and duration for each person-day unit.

- 1. **References**

1. MediaTek. MediaTek MT2502 for Wearables. *MediaTek* (2019) Available at: https://www.mediatek.com/products/wearables/mt2502 [Accessed July 8, 2019]

2. Quectel. Quectel GPS L70. (2019) Available at: https://www.quectel.com/product/l70.htm [Accessed July 8, 2019]

3. Mangold KA, Manson RU, Koay ESC, Stephens L, Regner M, Thomson RB, Peterson LR, Kaul KL. Real-Time PCR for Detection and Identification of Plasmodium spp. *J Clin Microbiol* (2005) **43**:2435–2440. doi:10.1128/JCM.43.5.2435-2440.2005

4. Singh B, Bobogare A, Cox-Singh J, Snounou G, Abdullah MS, Rahman HA. A genus- and species-specific nested polymerase chain reaction malaria detection assay for epidemiologic studies. *Am J Trop Med Hyg* (1999) **60**:687–692.

5. McLean DJ, Skowron Volponi MA. trajr: An R package for characterisation of animal trajectories. *Ethology* (2018) **124**:440–448.

6. Kareiva PM, Shigesada N. Analyzing insect movement as a correlated random walk. *Oecologia* (1983) **56**:234–238. doi:10.1007/BF00379695

1. **Supplementary Tables:**

**Supplementary Table 1. Baseline characteristics of study population**

|  | **Enrolled** | | **Non enrolled** | | **Overall** | | |
| --- | --- | --- | --- | --- | --- | --- | --- |
|  | **(n=20)** | | **(n=30)** | | **(n=50)** | | |
| **Travel in previous month** | |  | |  | | |  |
| Yes | 16 (80.0%) | | 23 (76.7%) | | 39 (78.0%) | | |
| No | 4 (20.0%) | | 7 (23.3%) | | 11 (22.0%) | | |
| **Sex ^** |  | |  | |  | | |
| Female | 5 (25.0%) | | 17 (56.7%) | | 22 (44.0%) | | |
| Male | 15 (75.0%) | | 13 (43.3%) | | 28 (56.0%) | | |
| **Age** |  | |  | |  | | |
| Mean (SD) | 40.1 (16.4) | | 38.8 (18.3) | | 39.3 (17.4) | | |
| **Occupation** |  | |  | |  | | |
| in-community | 6 (30.0%) | | 16 (53.3%) | | 22 (44.0%) | | |
| out-community | 14 (70.0%) | | 14 (46.7%) | | 28 (56.0%) | | |
| **Born in another village** |  | |  | |  | | |
| Yes | 8 (40.0%) | | 10 (33.3%) | | 18 (36.0%) | | |
| No | 12 (60.0%) | | 20 (66.7%) | | 32 (64.0%) | | |
| **Infection status** |  | |  | |  | | |
| Infected | 12 (60.0%) | | 18 (60.0%) | | 30 (60.0%) | | |
| Non-infected | 8 (40.0%) | | 12 (40.0%) | | 20 (40.0%) | | |
| **Education level^** |  | |  | |  | | |
| Primary or less | 19 (63.3%) | | 19 (95.0%) | | 38 (76.0%) | | |
| Secondary complete or incomplete | 9 (30.0%) | | 0 (0%) | | 9 (18.0%) | | |
| Superior | 2 (6.7%) | | 1 (5.0%) | | 3 (6.0%) | | |
| **Literate** |  | |  | |  | | |
| Yes | 23 (76.7%) | | 17 (85.0%) | | 40 (80.0%) | | |
| No | 7 (23.3%) | | 3 (15.0%) | | 10 (20.0%) | | |
| **Monthly income (PEN)** |  | |  | |  | | |
| Mean (SD) | 582 (796) | | 415 (255) | | 505 (604) | | |
| ^ Statistically significant differences to general population >18y. Fisher's exact test p-value<0.05. | | | | | |  |  |

1. **Supplementary Figures:**

**Supplementary Figure 1.** 3D Preview of the GPS-tracker CAD files corresponding to the 6 iterations of the case design.


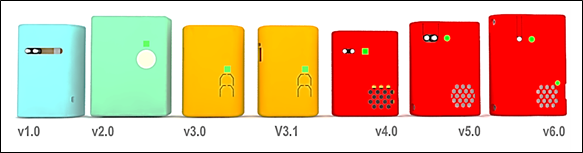


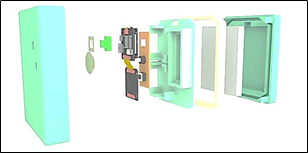

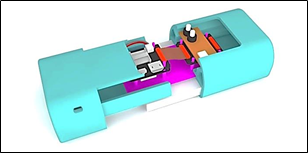


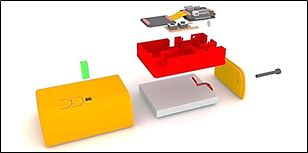

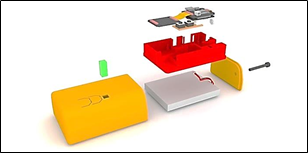


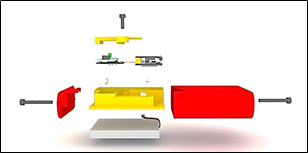

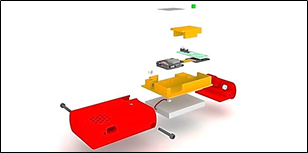


**
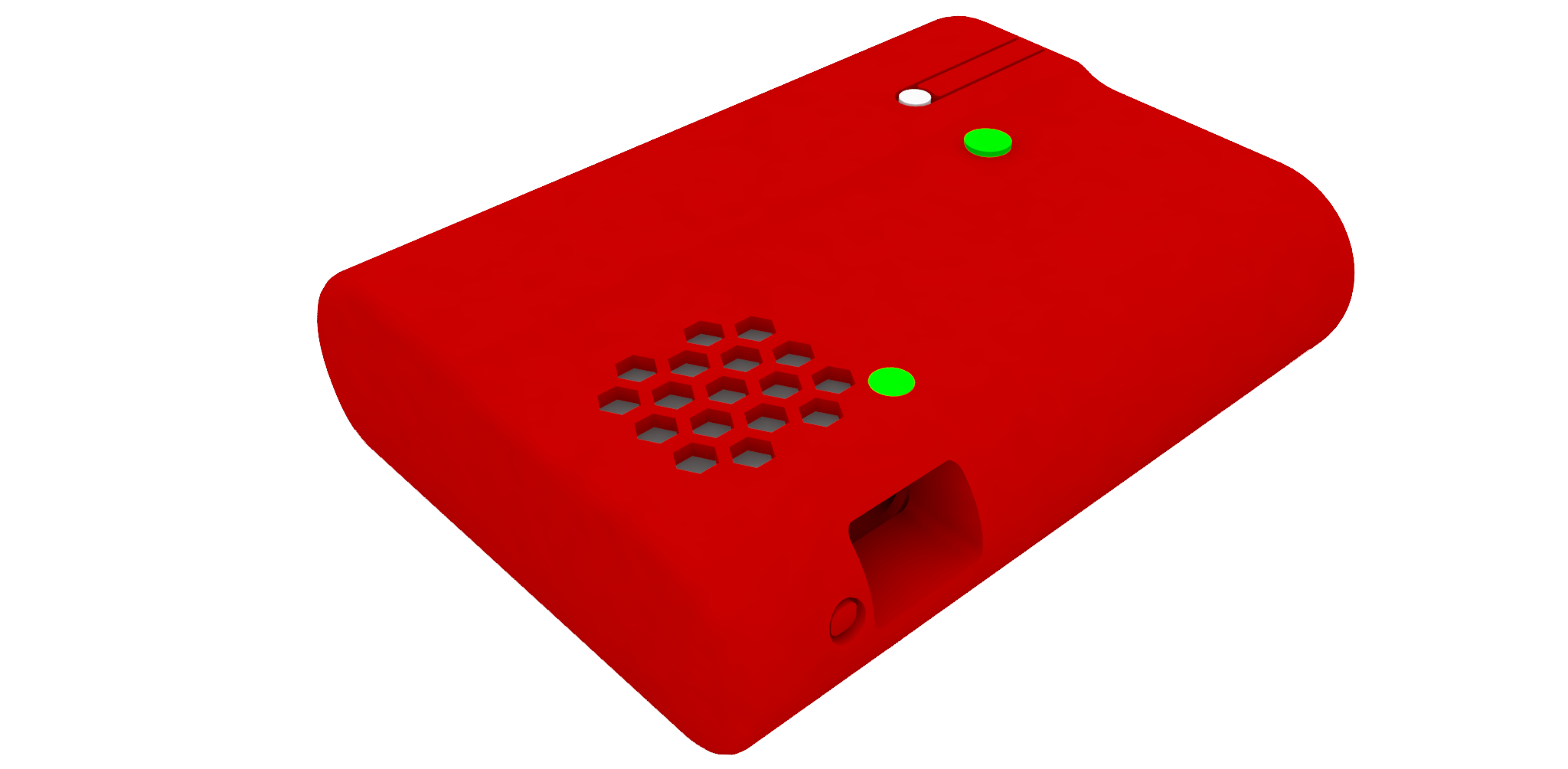
**

**Supplementary Figure 2.** Infection status aligned to trajectories timestamps at A) fixed and B) free spatial scales. Each box represents the participant’s movement.


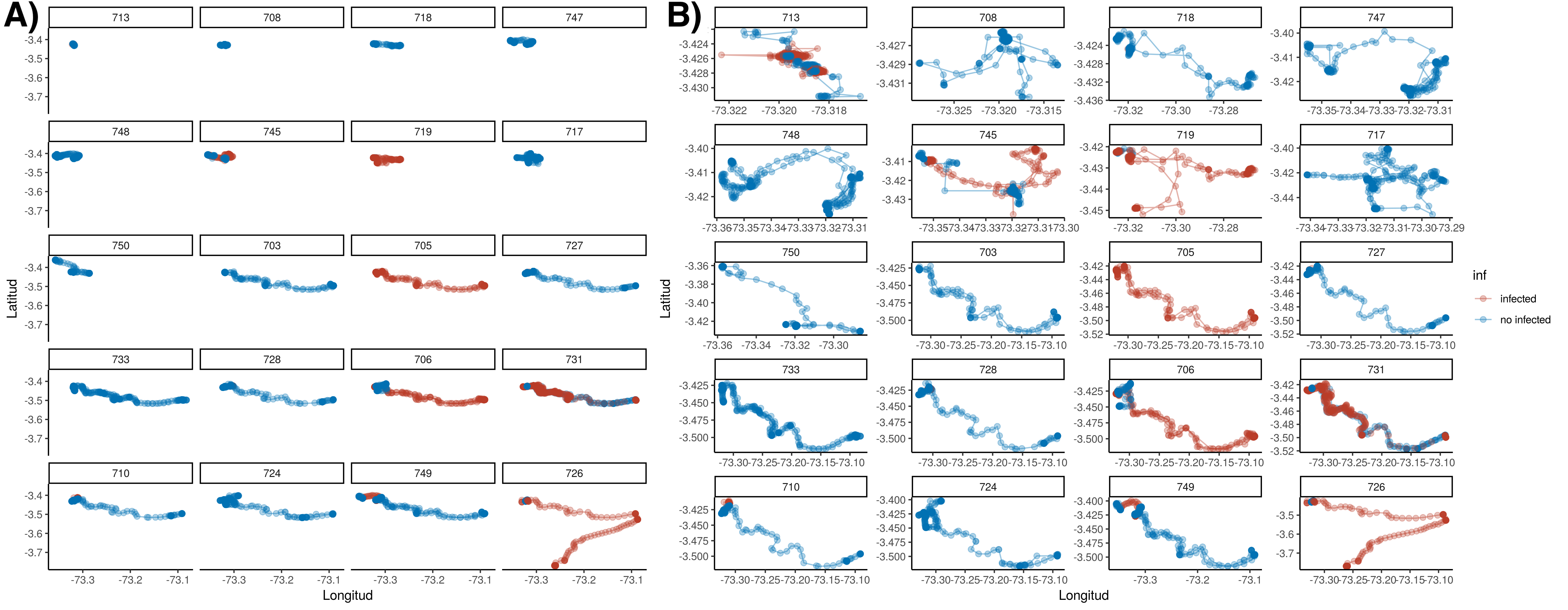

Supplement: Supplementary file 1 [file Data_Sheet_1.docx]
